# Major Group-B Enterovirus populations deleted in the noncoding 5’ region of genomic RNA modulate activation of the type I interferon pathway in cardiomyocytes and induce myocarditis

**DOI:** 10.1371/journal.ppat.1012125

**Published:** 2024-05-02

**Authors:** Domitille Callon, Marie Glenet, Anne-Laure Lebreil, Laetitia Heng, Nicole Bouland, Caroline Fichel, Paul Fornes, Laurent Andreoletti, Fatma Berri

**Affiliations:** 1 University of Reims Champagne Ardennes, Inserm, UMR-S1320 CardioVir, Reims, France; 2 Academic Hospital of Reims, Robert Debré, Pathology Department, Reims, France; 3 Academic Hospital of Reims, Robert Debré, Virology Department, Reims, France; University of Maryland at College Park: University of Maryland, UNITED STATES

## Abstract

Major 5’-terminally deleted (5’TD) RNA forms of group-B coxsackievirus (CVB-5’TD) has been associated with myocarditis in both mice and humans. Although it is known that interferon-β (IFN-β) signaling is critical for an efficient innate immune response against CVB-induced myocarditis, the link between CVB-5’TD RNA forms and type I IFN signaling in cardiomyocytes remains to be explored. In a mouse model of CVB3/28-induced myocarditis, major early-emerging forms of CVB-5’TD RNA have been characterized as replicative viral populations that impair IFN-β production in the heart. Synthetic CVB3/28 RNA forms mimicking each of these major 5’TD virus populations were transfected in mice and have been shown to modulate innate immune responses in the heart and to induce myocarditis in mice. Remarkably, transfection of synthetic viral RNA with deletions in the secondary structures of the 5’-terminal CVB3 RNA domain I, modifying stem-loops “b”, “c” or “d”, were found to impair IFN-β production in human cardiomyocytes. In addition, the activation of innate immune response by Poly(I:C), was found to restore IFN-β production and to reduce the burden of CVB-5’TD RNA-forms in cardiac tissues, thereby reducing the mortality rate of infected mice. Overall, our results indicate that major early-emerging CVB3 populations deleted in the domain I of genomic RNA, in the 5’ noncoding region, modulate the activation of the type I IFN pathway in cardiomyocytes and induce myocarditis in mice. These findings shed new light on the role of replicative CVB-5’TD RNA forms as key pathophysiological factors in CVB-induced human myocarditis.

## Author summary

Myocarditis is an inflammatory disease of the heart. Viruses or post-viral immune mediated response are the main causes of myocarditis. Human group B enteroviruses, specifically Coxsackievirus B3 (CVB3), are common infectious causes of acute myocarditis in the young. Early emergence and maintenance of major 5’terminally deleted RNA forms of CVB have been associated with acute and chronic myocarditis in both mice and humans. To achieve viral genome maintenance in the cardiac cells, viruses could modulate innate immunity activation. Here, we report on major early-emerging CVB3 populations deleted in the 5’non-coding region of viral genomic RNA that modulate the activation of the type I interferon pathway in cardiomyocytes and cause myocarditis in mice. Activating the innate immune sensors after CVB3 infection protects mice from CVB3-induced myocarditis and shall promote further studies in immunotherapies in viral acute and chronic myocarditis.

## Introduction

Group-B Enteroviruses (EV-B) are common and ubiquitous human pathogens transmitted through faecal-oral or respiratory routes. Although most EV-B infections remain asymptomatic, group-B coxsackieviruses (CVB) serotypes 1–6 are common causes of severe acute infection in children and young adults, such as myocarditis, pancreatitis or meningitis [1]. Acute EV-B infections can evolve toward persistent infections responsible for chronic pathologies such as dilated cardiomyopathy (DCM), type 1 diabetes and inflammatory myopathy [2–4].

EV-B are non-enveloped viruses, with single-stranded positive-sense RNA genome of approximately 7,400 nucleotides [1]. EV-B RNA genome is flanked on the 5’ end by a conserved untranslated region (5’UTR) which is crucial for initiation of viral replication and translation activities [1]. A Coxsackievirus-B2 harboring 5’terminally deleted (CVB-5’TD) population ranging from 22 to 36 nucleotides (nt) was found in the heart of a patient who died of fulminant myocarditis [5]. Since 2016, our team identified by next generation sequencing (NGS) or rapid amplification of cDNA-end by polymerase chain reaction (RACE-PCR) assays major EV-B populations characterized by 5’UTR deletions ranging from 8 to 50nt (EVB-5’TD), either alone or associated with low proportions of full-length viruses in acute myocarditis or DCM human samples [6–8]. Similarly, major CVB-5’TD populations have been found in persistent cardiac and pancreatic infections in immunocompetent mouse models [9,10]. These EVB-5’TD populations were characterized by a decrease in ratios of positive to negative [(+)/(−)] RNA strands, and low viral protein synthesis activities [6,8,10,11]. Taken together these clinical and experimental findings suggest that these replicative EVB-5’TD populations are associated with the development of acute or chronic myocarditis in humans and mouse models [10,12,13].

Reported deletions within the 5’UTR of EV-B forms are known to affect functional secondary-structural elements of the RNA domain I (cloverleaf) [11,14,15]. Leveque et al. demonstrated a reduced genomic replicative capacity of these EVB-5’TD RNA forms which was explained by a disturbed binding of cellular factor Poly(rC)-binding protein 2 and viral factor polymerase 3CD on the 5’UTR domain I secondary structure [11]. Viral genome maintenance is a key step to develop acute phase of infection and to establish viral RNA persistence in cardiac cells. To achieve viral genome maintenance and long-lasting detection of EV-B in the heart, low-levels replication and translation activities of naturally produced 5’TD viruses might be associated with the capacity to modulate innate immunity activation [16,17].

The innate immune response mediated by type I interferon (IFN) is known to be crucial in the progression of acute viral myocarditis, which may evolve toward either healing or chronic persistent infection [16,18]. This crucial role is evidenced by the variety of mechanisms used by RNA(+) viruses to evade or inhibit IFN induction, signalling or effector functions [19–21]. Type I IFN induction has been shown to protect against EV infection in various models [20,22,23]. However, a moderate type I IFN response has been shown to promote persistent viral infections [24,25]. It was reported that in a CVB3 mouse model, a reduced type I IFN response resulted in worsening of myocarditis [26]. Deletions into the 5’UTR of EV-B populations could modify the RNA secondary-structural elements recognized by innate immune sensors such as RIG-I-like-receptors (RLR, such as Retinoic-inducible gene I, RIG-I or Melanoma differentiation-associated protein 5, MDA5) during EV-B acute infections, resulting in a modulation of viral RNA sensing and type I IFN signalling pathway activation [12,27,28]. We recently showed that cardiac and pancreatic acute CVB3/28 infections in immunocompetent mice resulted in a low interferon-β (IFN-β) response [10]. Although it is known that IFN-β signaling is critical for an efficient innate immune response against CVB-induced myocarditis, the relationship between CVB-5’TD RNA forms and type I IFN signaling in cardiomyocytes remains to be explored.

In the present report, we first analysed the impact of early emergence of replicative CVB-5’TD RNA forms on cardiac type I IFN levels during a CVB3-induced myocarditis in an immunocompetent DBA/2J mouse mode. Next, synthetic major CVB-5’TD RNA forms were transfected into DBA/2J mice to investigate the direct role of CVB-5’TD RNA forms on the modulation of IFN-β pathway activation and myocarditis pathogenesis. Subsequently, synthetic CVB3/28 RNA forms mimicking early emerging 5’terminal deletions were transfected in cultured human cardiomyocytes to explore the regulatory effect of major viral 5’ RNA-domain I deletions on type I IFN pathway induction. Finally, CVB3-infected DBA/2J mice were treated with poly(I:C), an agonist of TLR3 (Toll-like-receptors) and RLR (RIG-I like receptors), to restore an innate immune response. Overall, our experimental results indicate that major CVB-3 populations deleted in the 5’ noncoding region in domain I of genomic RNA modulate activation of the type I IFN pathway in cardiomyocytes and induce myocarditis in mice.

## Results

### Early emergence of 5’terminally deleted CVB populations modulates type I interferon response

**Fig 1:**
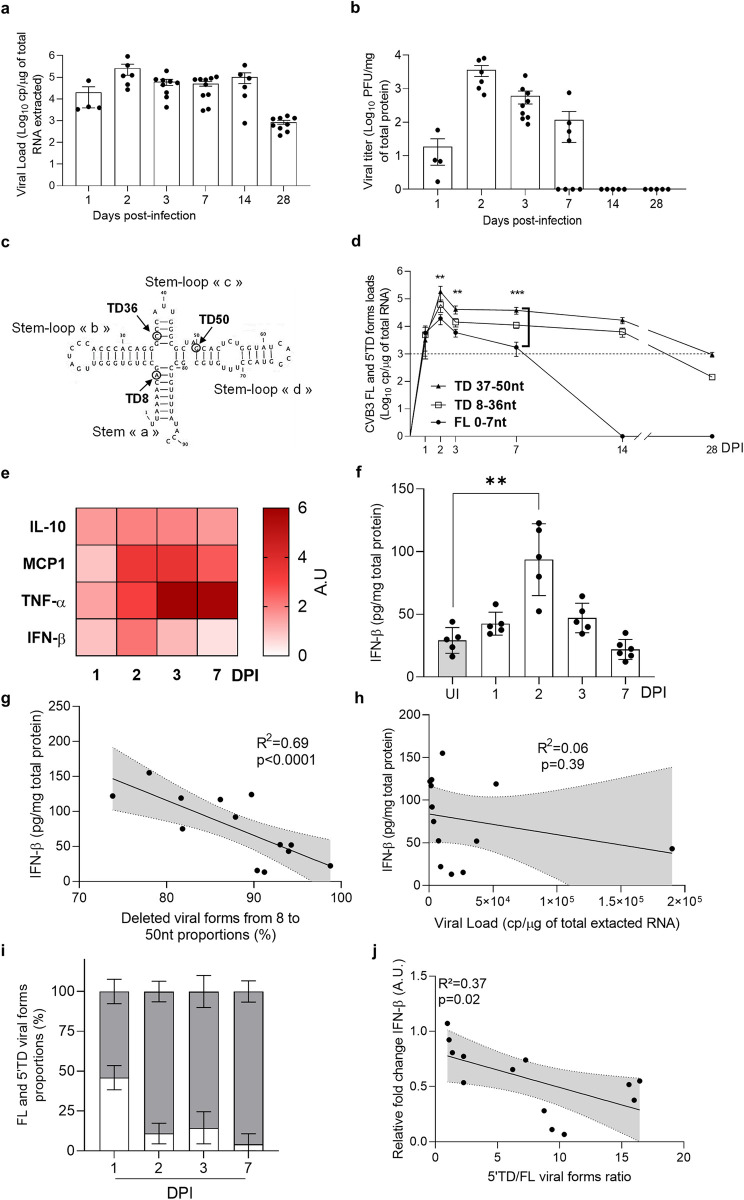
The emergence of 5’terminally deleted (5’TD) CVB populations is associated with a low type I interferon response in the heart. **a** Viral load levels (RT-qPCR) in the heart of infected mice at 1, 2, 3, 7, 14- and 28-days post-infection (DPI) (n = 4 to 9). **b** Viral titers (PFU) in the homogenized heart supernatants at 1, 2, 3, 7, 14- and 28 DPI (n = 4 to 9). **c.** Two-dimensional representation of the EV-B 5’non-coding region RNA sequences showing the position of the CVB3/28 5’ terminally deleted forms. **d.** CVB-5’TD and FL viral loads were assessed using a RACE-PCR method associated with a micro-electrophoresis, the profile of CVB-TD forms distribution was confirmed by NGS method, at 1, 2, 3, 7, 14- and 28-DPI (n = 4 to 9). Data represent mean ±SD. (Two-way ANOVA Multiple comparisons; **: p<0.01; ***: p<0.001). **e** Cytokines levels measured by ELISA (heatmap view) in homogenized heart supernatants expressed as fold change relative to uninfected mice data (n = 6). **f** IFN- β levels in the homogenized heart supernatants quantified by ELISA at indicated time points post-infection. **g** Linear regression curves and Spearman R coefficient of correlation between IFN-β levels and the proportions of 8-50nt 5’TD viral populations (2–3 DPI, n = 13). **h** Linear regression curves and Spearman R coefficient of correlation between IFN-β levels and total viral load (2–3 DPI, n = 13). **i** Full-length (white) and 5’terminally deleted (grey) viral forms proportions in hearts of CVB3/28 infected mice from 1 to 7 DPI. **j** Linear regression curves and Spearman R coefficient of correlation between cardiac IFN-β mRNA fold-changes and the 5’TD/FL ratio (24 to 72 HPI; n = 13). 5’TD: 5’terminally deleted; FL: full length; UI: uninfected.

To investigate the dynamics of 5’terminally deleted (CVB-5’TD) viral population’s emergence in mice, we first quantified total viral RNA loads in cardiac tissues of CVB3/28 infected mice (Fig 1A). Viral RNA loads in the heart exhibited an increase until 2 days post-infection (DPI) (Fig 1A) and then a decrease until 28 DPI but remained detectable with significant mean values of 10^3^ cp/μg of cardiac tissues (Fig 1A). Progeny viral production was quantified by classical plaque forming unit assay (PFU), demonstrating a peak at 2 DPI with an absence of detectable infectious particles after 14 DPI, corresponding to the beginning of the viral persistent phase in cardiac tissues (Fig 1B and Table 1). Positive/negative-strand RNA ratio was high at 3 DPI, and low at 14 and 28 DPI, similarly to single-strand/double-strands RNA ratio (Table 1). Encapsidation of viral RNA, demonstrated by a viral entry challenge with or without proteinase K, was maintained at all times post infection (Table 1). Using a RACE-PCR assay followed by a previously validated RNA sequencing strategy by NGS we specifically quantified the proportions of each 5’TD and full-length (CVB-FL) viral populations at different DPI in cardiac tissues. Two major groups of CVB-5’TD populations were characterized: one with deletions ranging from 8 to 36 nucleotides (nt), with a partial or total loss of stem-loop “b”; and one with deletions ranging from 37 to 50nt, characterized by a partial or total loss of stem-loop “c” and a partial loss of stem-loop “d” (Figs 1C and 1D and S1A). These CVB-5’TD populations, significantly major after 2 DPI, were initially associated with CVB-FL that became undetectable after 14 DPI (Fig 1C and 1D). Our results demonstrated an early emergence of two major CVB-5’TD population’s groups during acute myocarditis without any detectable CVB-FL forms after 14 DPI, corresponding to the subacute phase of the myocarditis.

**Table 1:** Viral properties of detected CVB3 populations at 3, 14 and 28 DPI.

		Heart		
	Days Post Infection	3	14	28
	**(+)/(-) vRNA Ratio**	59.8	5.0	4.8
	**ss/ds vRNA Ratio**	39.4	7.0	0.4
**Viral cell-culture challenge (HeLa229 cells, 72-h)**	**Entry Capacity (%)**	68	24	48
	**Proteinase K-treated Entry Capacity (%)**	0	0	0
	**Fold of normalized genome copy increase**	587	2.5	12.1
	**PFU (PFU/ml)**	3*10^7^	ND	ND

N = 3; (+)/(-) or ss/ds vRNA Ratio: positive/negative or single/double-strands viral RNA Ratio. PFU: Plaque forming unit. ND: non-detectable.

To assess the innate immune response after CVB3/28 infection in mice, we first validated our DBA/2J mice model for type I IFN production upon a prototype viral RNA infection. Two Mengovirus strains that are known for impairing (WT) and inducing (Zn) IFN-β production levels in brain and blood, and Poly(I:C) were inoculated in DBA/2J mice [29]. As shown in S1B Fig, a significant increase in IFN-β protein levels was observed in CVB3/28-infected and Poly(I:C)-inoculated mice at 2 DPI as well as in Mengovirus Zn-infected mice at 3 DPI. As expected, Mengovirus WT-infected mice did not experience an increase in IFN-β protein secretion (S1 Fig). Following an intraperitoneal CVB3/28 infection in DBA/2J mice, cytokine levels (MCP-1, IL-10, TNF-α, IFN-β) were quantified in homogenized cardiac tissues at different time points post-infection. By contrast with dynamics of other tested cytokines, IFN-β protein levels significantly dropped at 3 DPI after a slight increase at 2 DPI (Fig 1E and 1F). Remarkably, IFN-β protein levels appeared to be negatively correlated with proportions of 8-50nt CVB-5’TD forms detected between 2 and 3 DPI (R^2^ = 0.69, p<10^−3^) (Fig 1G), while no positive or negative correlation was observed with total viral RNA loads measured in cardiac tissues (Fig 1H). Moreover, no correlation was found between proportions of 8-50nt CVB-5’TD forms and others cytokines (S2 Fig). As expected, CVB-5’TD RNA forms proportions experienced a significant increase between 1 and 2, 3 and 7 DPI (Fig 1I), and IFN-β mRNA fold-changes were negatively correlated with CVB-5’TD/FL ratio from 2 to 3 DPI (R^2^ = 0.37; p = 0.02) (Fig 1J). Altogether these findings suggest that CVB-5’TD forms could modulate type I IFN pathway activation in cardiac tissues during the early stage of the CVB3 infection, promoting the development of acute and subsequently chronic myocarditis phases.

### Inoculation of cardiac CVB-5’TD populations in mice induces myocarditis and modulates IFN-β production

**Fig 2:**
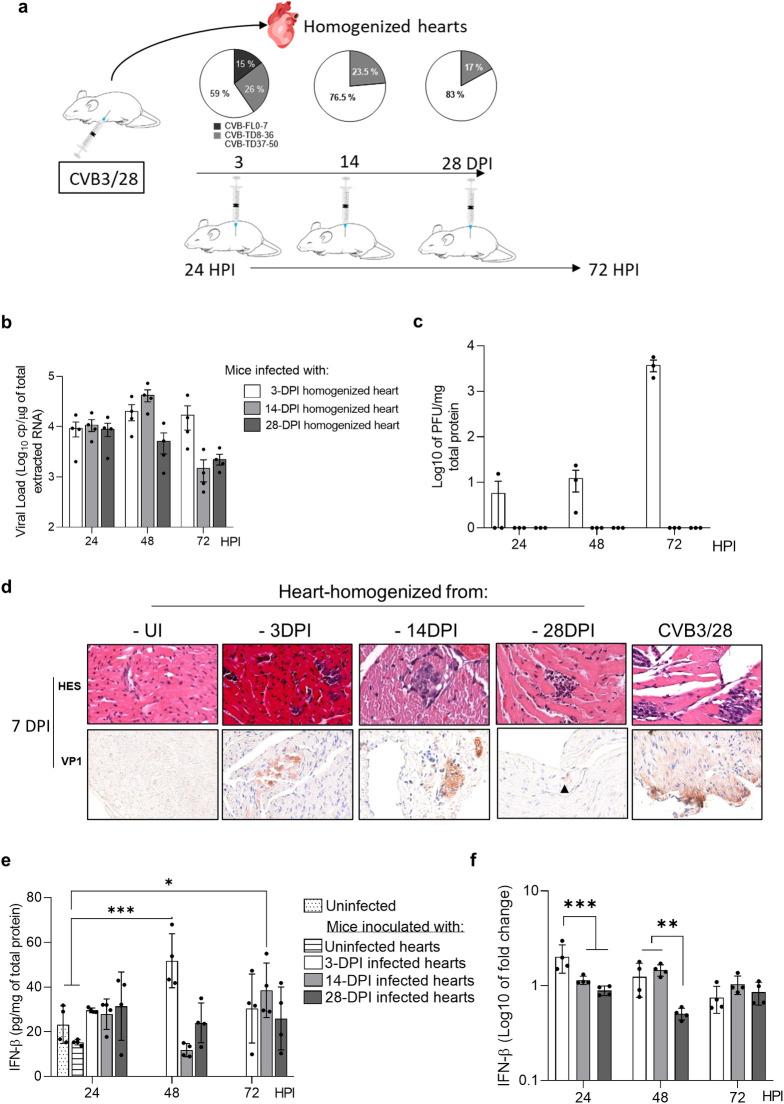
Inoculation of cardiac CVB RNA populations in mice induces acute and chronic myocarditis. **a** Schematic illustration of the experimentation. **b** Viral loads (RT-qPCR) in the hearts of mice infected with homogenized hearts or CVB3/28 (n = 4). **c** Viral titers (PFU assay) in the hearts of mice infected with homogenized hearts or CVB3/28. **d** Histology (HES) and immunohistochemistry for viral protein 1 (VP1, orange-brown staining) of mouse hearts at 7 DPI. **e, f** IFN-β cytokine and mRNA levels fold-changes (ELISA and RT-qPCR) in homogenized hearts supernatants of mice infected with homogenized hearts or CVB3/28 (n = 4) at the indicated points time post-infection. Data represent mean ± SD (Two-way ANOVA Multiple comparisons; *: p<0.05; **: p<0.01; ***: p<0.001 *versus* CVB3/28-infected mice or uninfected mice). UI: uninfected.

To assess CVB-5’TD populations’ ability to infect cardiac cells *in vivo*, we inoculated DBA/2J mice with homogenized-hearts collected from previously 3 (15% of CVB-FL; 26% of CVB-5’TD 8-36nt and 59% of CVB-5’TD 37-50nt), 14 (23.5% of CVB-5’TD 8-36nt; 76.5% of CVB-5’TD 37-50nt) or 28 (17% of CVB-5’TD 8-36nt and 83% of CVB-5’TD 37-50nt) days post-CVB3-infected mice (Fig 2A). Following an infection running from 24 to 72 hours, we showed that the inoculation of cardiac CVB-5’TD populations from 3, 14 and 28 DPI-infected mice resulted in active viral infections in the heart (Fig 2B). Despite the absence of FL RNA forms in 14 and 28 DPI homogenized-hearts supernatants (Fig 2C), their intra-peritoneal inoculation induced sparse inflammatory foci, VP1 positive detection in cardiac cells and inflammatory cells’ infiltrates (Figs 2D and S3). Inoculated CVB3-5’TD populations remained detectable until 42 DPI, indicating a persistent infection (S3A Fig). Mice inoculated with 3-DPI homogenized-hearts experienced a significant increase in IFN-β protein and mRNA levels at 48 HPI (Fig 2E and 2F). IFN-β production was not or less increased in mice infected with 14 and 28-DPI homogenized hearts, in comparison with those infected with 3-DPI homogenized hearts. Together, these results show that naturally emerging cardiac CVB-5’TD populations can infect and replicate in mice cardiac tissues following an intraperitoneal inoculation, modulating IFN-β production and inducing myocarditis despite the absence of CVB-FL and detectable infectious viral particles.

### *In vivo* transfection of CVB-5’TD RNA forms induces myocarditis without an IFN-β increase

**Fig 3:**
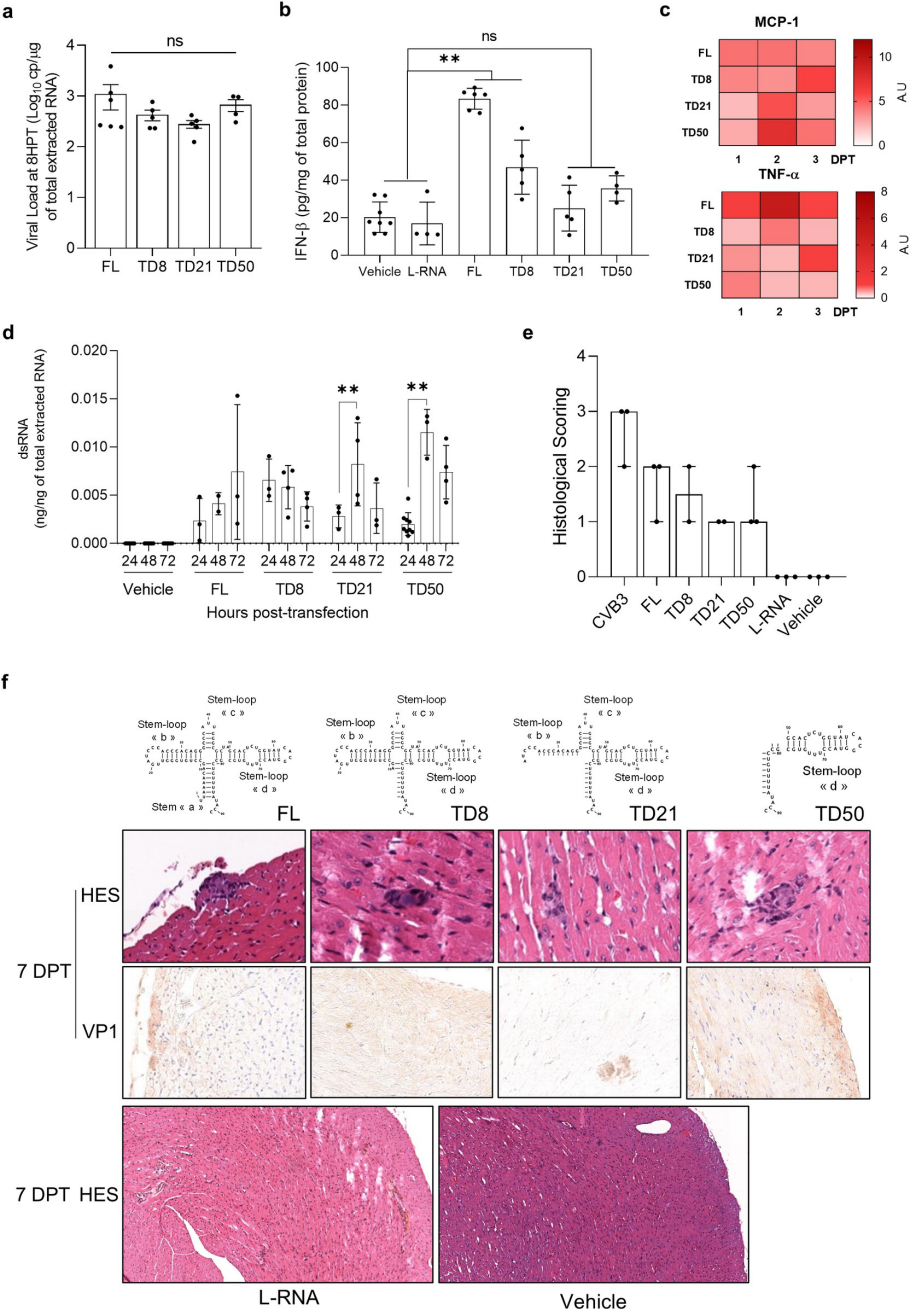
Major CVB-5’TD RNA forms transfection in DBA/2J mice induces acute myocarditis without IFN-β increase. **a** Viral load (RT-qPCR) in the hearts of mice transfected with CVB-FL, 5’TD8, 5’TD21 or 5’TD50 RNA forms, at 8 hours post transfection (n = 4 to 6). **b** IFN-β cytokine levels (ELISA) in hearts of uninfected mice or mice transfected with vehicle alone, long-RNA, CVB-FL, 5’TD8, 5’TD21 or 5’TD50 RNA forms, at 8 hours post transfection. Data represent mean ± SEM (n = 4 to 8) (Two-way ANOVA Multiple comparisons; **: p<0.01; ns: non-significant; *versus* vehicle transfected mice). **c** Cytokines levels measured by ELISA (heatmap view) in homogenized heart supernatants expressed as fold change relative to uninfected mice data (n = 2 to 6). **d** Double-strands RNA quantification by ELISA assay in cardiac lysates between 24 and 72 hours post transfection. (n = 2 to 9) (Mann-Whitney U test: **: p<0.01). **e** Histological scoring of necrosis and inflammatory infiltrates in 3 slices per condition. **f** Histology (HES) and immunohistochemistry for viral protein 1 (VP1, orange-brown staining, black arrows) of mouse hearts at 7 DPI (n = 3). DPT: days post-transfection; UT: untransfected mice; TD: terminally deleted forms; VP1: Viral protein 1.

To better understand the direct link between CVB-5’TD RNA forms and IFN-β modulation, we performed *in vivo* transfection assays of CVB-FL and 5’TD viral RNA forms. Using Jet-PEI reagent *in vivo*, by intravenous route, we show that all synthetic RNA forms are detectable in the heart 8 hours after transfection with similar replication activities levels (Fig 3A). We show that CVB-FL and CVB-5’TD8 RNA forms transfection induced a significant IFN-β increase in the heart (p = 0.008, p = 0.03 respectively) (Fig 3B). By contrast, CVB-5’TD21 and CVB-5’TD50 RNA forms transfection did not induce any significant variations in IFN-β levels by comparison with the vehicle-transfected-mice (Fig 3B). Interestingly, no significant differences were observed in the production of other inflammatory cytokines (CCL2/MCP-1 and TNF-α) after transfection with different 5’TD forms, at different time post-transfection (Fig 3C). The induction of inflammatory response by the 5’TD forms could be explained by an active viral genomic replication early after transfection. Using dsRNA ELISA kit, we observed an increase in replicative intermediates viral RNA at 48h post-transfection for TD21 and TD50 (Fig 3D). All transfected RNA forms induced inflammatory foci in the heart at 7 DPT, whereas a control Long-RNA did not (Fig 3E and 3F). Moreover, viral translation activities were assessed by VP1 immunohistochemistry assay in cardiac tissues (Fig 3F). CVB-FL and 5’TD-RNA forms transfection resulted in viral protein production in cardiac cells 7 days after transfection. These results demonstrate that CVB-5’TD21 and CVB-5’TD50 RNA forms transfection impair IFN-β production and induce myocarditis in an immunocompetent mice model, by contrast with FL and 5’TD8 RNA transfected forms.

### Impact of CVB-FL and CVB-5’TD forms transfection on IFN-β pathway activation in human cardiomyocytes

**Fig 4:**
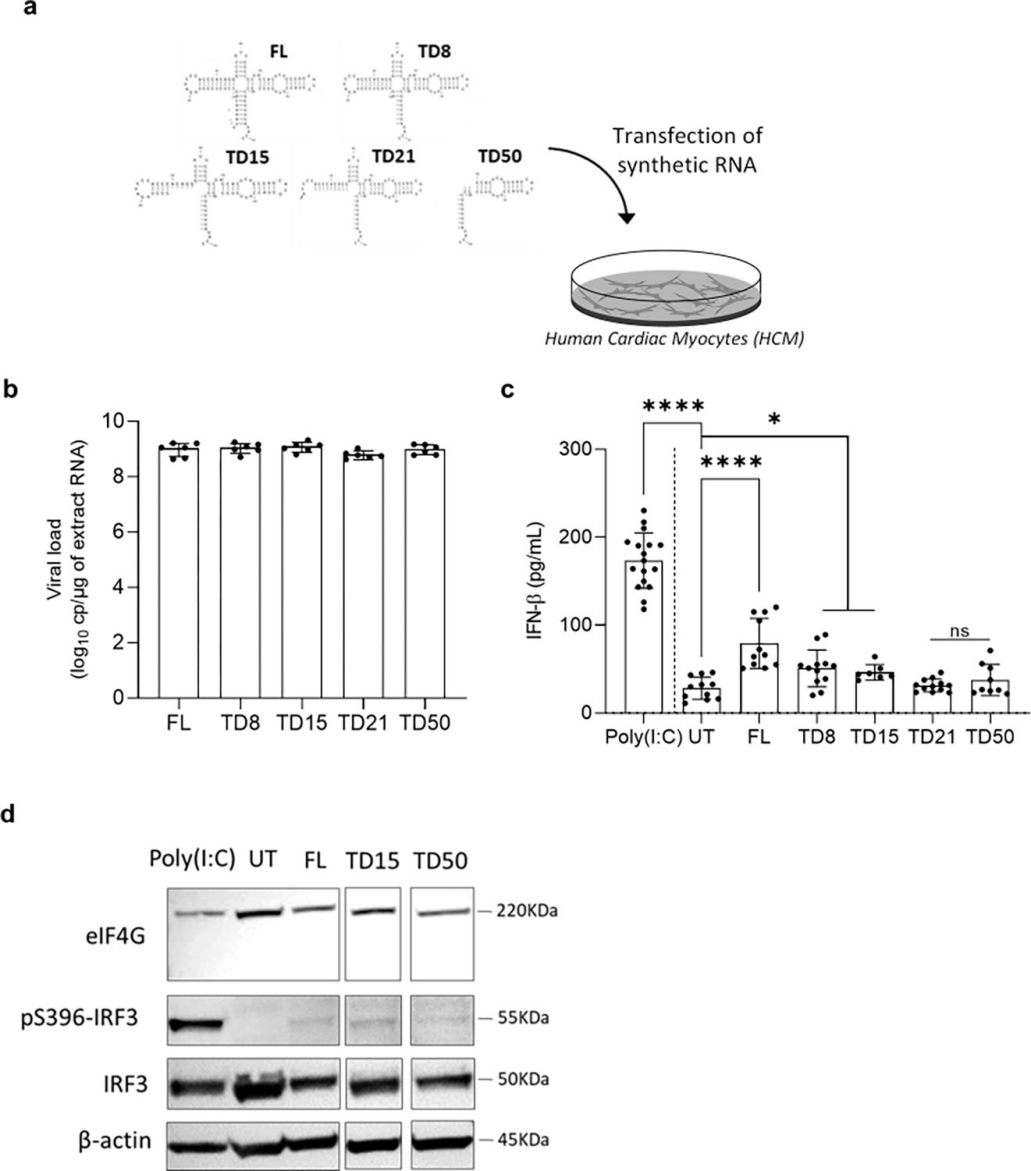
Impact of CVB3 viral populations on IFN-β pathway activation in cultured human cardiomyocytes. **a** Schematic illustration of the experimentation: transfection of CVB-FL and CVB-5’TD (5’TD8, 5’TD15, 5’TD21 and 5’TD50) synthetic RNA forms into human cardiac myocytes cells (HCM). **b** Viral load (RT-qPCR) in HCM transfected cells with CVB-FL and CVB-5’TD (5’TD8, 5’TD15, 5’TD21 and 5’TD50) synthetic RNA at 8 hours post-transfection. **c** IFN-β cytokine levels (ELISA) in supernatants of HCM at 8 HPT of various CVB-TD/or full-length RNA forms. **d** Analysis of eIF4G cleavage, and levels of phosphorylated IRF3 (pS386-IRF3) and IRF3 on a Western blot prepared with HCM cells collected at 8 HPT. Poly(I:C) is used as positive control of IFN-β pathway activation. Data represent mean ± SD (n = 3) (Mann-Whitney U test; *: p<0.05; ****: p<0.0001 and ns: non-significant). eIF4G: eukaryotic translation initiation factor 4 G; HMW: high molecular weight Poly(I:C); IRF3: Interferon Regulatory Factor 3; eIF4G: eukaryotic initiation factor 4 gamma; UT: untransfected.

Since major CVB-5’TD forms were associated with an impairment of IFN-β response in mice’s hearts, a key biological issue was to identify 5’UTR secondary-structural elements able to modulate innate immune response by transfecting CVB-FL and CVB-5’TD RNA forms into human primary cardiac myocytes cells (HCM) (Fig 4A). All synthetic CVB-5’TD RNA forms harbor a hammerhead ribozyme at their 5’termini, which generate an authentic 5’terminal CVB3 sequences explaining the absence of tri-phosphorylation at the extremity 5’ known to induce type I IFN response by specific RIG-I recognition [11]. After 8 hours post-transfection of each 5’TD RNA forms, a same amount of viral RNA load was quantified in transfected HCM (Fig 4B). Interestingly, the quantification of IFN-β cytokine in the supernatant of transfected HCM (Fig 4C), revealed an increase in IFN-β concentration after CVB-FL, CVB-5’TD8 and CVB-5’TD15 transfection but not with CVB-5’TD21 and CVB-5’TD50 (Fig 4C) comparatively to the untransfected cells. To assess their impact on type I IFN pathway activation, lysates of transfected HCM by 5’TD RNA forms (FL: non-deleted, TD15 (representative of a population with deletion between 8-36nt) and TD50 (representative of a population with deletion between 37-50nt)) were analyzed by immunoblot for eIF4G (eukaryotic initiation factor 4 gamma) cleavage and phosphorylation of IRF3 (Interferon Regulatory Factor 3). As shown in Fig 4D, the TD50 RNA form exhibited a very low phosphorylation of IRF3 compared to FL and TD50 RNA forms, independently of the cleavage of eIF4G. Viral proteases, such as protease 2A, are known to cleave eIF4G in human cells [8,30]. These results indicate that stem loops “b”, “c” or “d” of the viral RNA domain I are implicated in IFN-β signaling pathway induction in human cardiac cells and that the 5’ppp is not required for IFN induction.

### Poly(I:C)-induced IFN-β production decreases 5’terminally deleted CVB RNA replication in the heart of CVB3/28-infected mice

**Fig 5:**
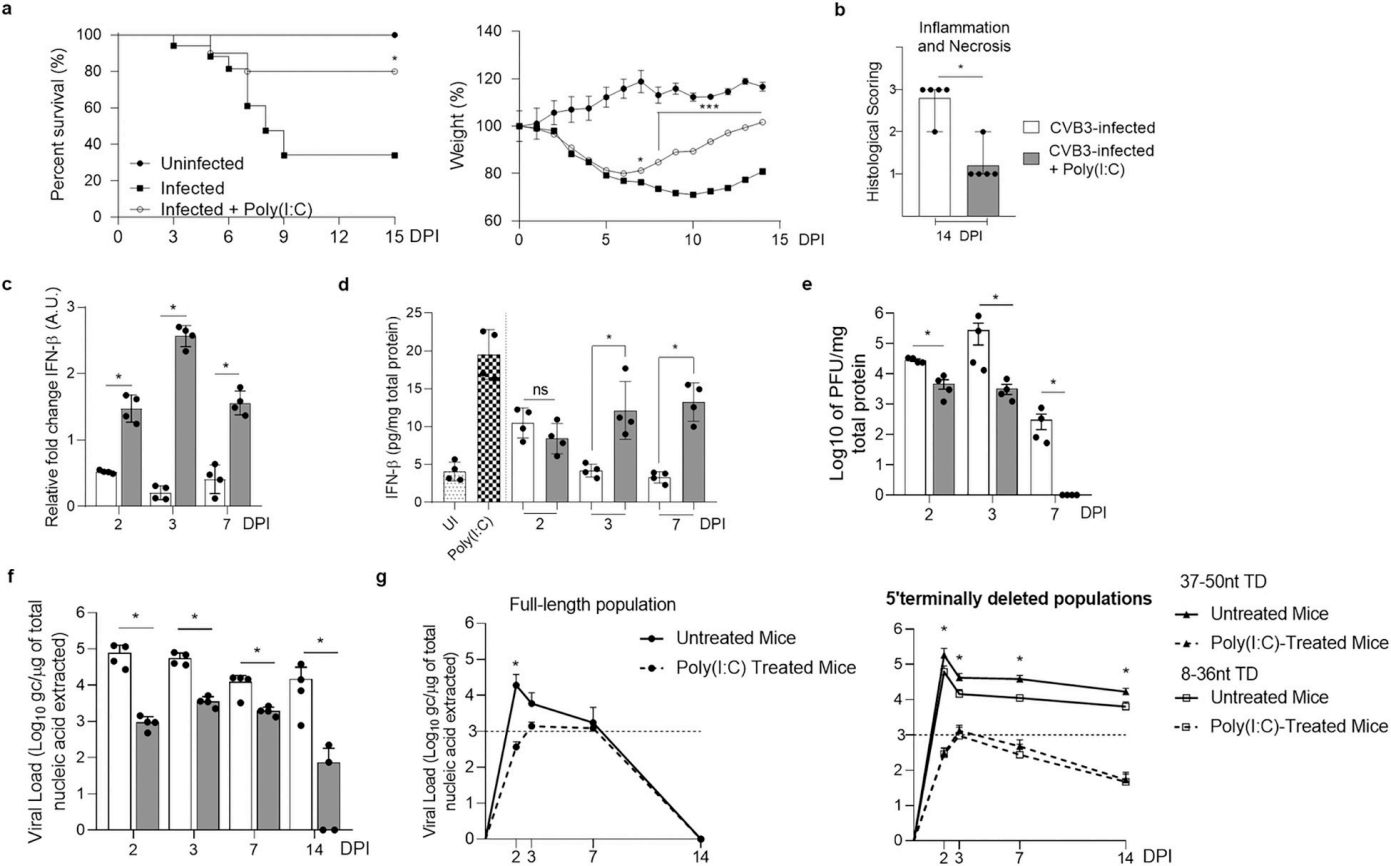
IFN-β signalling pathway stimulation by Poly(I:C) decreased 5’terminally deleted CVB3 RNA forms viral loads, cardiac lesions and mortality in DBA/2J mice. **a** Survival curve and follow-up of body weight of CVB3/28-infected mice (n = 17; black square) compared to infected Poly(I:C) HMW-treated mice (n = 10; white circle) and uninfected mice (n = 9; black circle) (*: p<0.05 by Log-Rank test between infected and infected-treated groups). Data represent mean ±SEM (*: p<0.05, ***: p<0.001, by Tukey’s multiple comparison test between infected and infected-treated groups). **b** Semi-quantification of inflammation and necrosis in treated group (grey bar) and untreated group of mice (white bar) (n = 5 each) at 14 DPI. Data represent mean ± range (Mann-Whitney U test, *: p<0.05). **c d** IFN-β mRNA fold-changes and ELISA of IFN-β in homogenized heart supernatants of treated and untreated infected mice (n = 4 each) (Mann-Whitney U test, *: p<0.05). **e f** Viral titers (PFU assay) and loads (RT-qPCR) of treated infected mice versus untreated infected mice (n = 4) (Mann-Whitney U test, *: p<0.05). **g** CVB-TD and FL respective viral loads were assessed as previously described for treated infected mice (n = 3 each DPI) and compared with untreated infected mice (n = 6 to 10). Data represent mean ± SD. (Two-way ANOVA Multiple comparisons; *: p<0.05; untreated mice *versus* Poly(IC)-treated mice). HMW: high molecular weight poly(I:C). DPI: days post-infection.

Since 5’TD CVB3 RNA forms proportions can impair IFN-β signaling pathway activation in our mouse myocarditis model, we postulated that the *in vivo* induction of high IFN-β production levels could significantly decrease CVB3-5’TD RNA forms loads in hearts of CVB3/28-infected mice. In order to explore this hypothesis, we used a synthetic dsRNA analogue (Poly(I:C)) known to induce type I IFN response *in vitro* and *in vivo* through TRL3 and RLR activation pathway [28,31]. Mice were treated by 150μg of Poly(I:C) for two consecutive days after a CVB3/28 intraperitoneal infection (1 and 2 DPI). Poly(I:C)-treated mice experienced a significant reduction in mortality rates (Log-Rank test; p = 0.004) and body-weight-loss in comparison with untreated mice (Tukey’s multiple comparisons test; p<0.014) (Fig 5A). In CVB3/28 infected mice, cardiac inflammation and necrosis levels were significantly lower in the Poly(I:C)-treated than in the untreated group (p = 0.016) (Fig 5B). IFN-β mRNA and protein levels in the heart were increased in treated mice in comparison with untreated mice, after 2 or 3 DPI respectively (p<0.029) (Fig 5C and 5D). Poly(I:C) treatment decreased viral RNA loads and PFU titers in the hearts from 2 to 14 DPI (p = 0.029) (Fig 5E and 5F). In Poly(I:C)-treated mice, 8-36nt and 37-50nt 5’TD CVB3 RNA load significantly decreased at 2, 3, 7 and 14 DPI comparatively to untreated mice (p<0.025) (Fig 5G). Overall, these results confirm that the type I IFN signaling pathway activation is a key-driver in the development of an efficient innate immune anti CVB response resulting in a significant viral clearance of CVB-5’TD populations, which can protect DBA/2J mice against severe CVB3/28-induced cardiac lesions.

## Discussion

The emergence of major 5’-terminally deleted RNA forms of group B coxsackievirus (CVB-5’TD) has been associated with myocarditis in both mice and humans [2–4,10,13,16,20,32]. While type I interferon (IFN) signaling is known to be critical for an efficient innate immune response against EV-B in mouse and human myocarditis, the relationship between CVB-5’TD RNA forms and type I IFN signaling in cardiomyocytes remains to be explored. In a mouse model of CVB3/28-induced myocarditis, we evidenced a major early-emerging replicative CVB-5’TD RNA forms impairing IFN-β production in cardiac tissues. Synthetic CVB3/28 RNA forms mimicking each of these major 5’TD virus populations were transfected alone into mice, modulating innate immune responses in cardiac tissues, and resulting in myocarditis. Deletions in the secondary structures of the 5’terminal CVB3 RNA domain I were found to impair IFN-β production in human cardiomyocytes transfected with synthetic viral RNA. In addition, the poly(I:C)-treatment of infected mice, restored the IFN-β production and reduced the burden of CVB-5’TD RNA -forms in cardiac tissue, thereby decreasing the mortality rate. Overall, our findings demonstrated how natural deletions within the 5’uncoding region affecting the secondary-structural elements of the genomic RNA domain I (cloverleaf structure) modulate type I IFN signaling pathway in cardiomyocytes, promoting the development of acute and chronic CVB3-induced myocarditis of immunocompetent mice.

In the present report, we investigated dynamics of emergence of CVB-5’TD and full-length (CVB-FL) populations in mice cardiac tissues. RNA-dependent RNA polymerase errors and lack of proofreading are likely to explain early generation of CVB-5’TD forms in target cells (Fig 1C) [33]. In the heart of CVB3-infected mice, emerging CVB-5’TD populations became major after 48 hours of infection (HPI), in comparison with CVB-FL forms, the minority population thereafter (Fig 1C). These results suggested an evolutionary advantage of CVB-5’TD forms in specific host cell conditions. Recently, we identified that these cardiac early-emerging CVB-5’TD populations associated with CVB-FL forms, were characterized by significant viral genomic replication activities [10,34]. The 37-50nt 5’terminal deletions could reduce the binding of specific replication factors to negative-strand RNA [35,36]. Loss of such RNA binding might contribute to the significant reduction of positive-strand RNA synthesis and aberrant low **(+)/(-)** RNA ratios found in CVB3-infected mouse hearts [10,36,37]. Consequently, hijacking of viral or cellular host factors of ribonucleoprotein complexes (RNP) by major levels of CVB-5’TD forms could significantly impair genomic replication activities of minor CVB-FL forms explaining why they are undetectable from 14 to 28 DPI in the heart (Fig 1C). Overall, our results suggest that specific host-cell conditions are necessary for emergence and maintenance of CVB-5’TD populations in mouse hearts [10,11,36,38].

These viral RNA are known to be recognized by innate immune sensors such as RIG-I-like-receptors (RLR) resulting in IFN-β signaling pathway activation [15,27]. Despite high levels of these replicative viral RNA forms, no strong nor sustained IFN-β pathway activation was observed, revealing a potential immunomodulatory effect of these CVB-5’TD populations (Fig 1E–1G). In comparison, Mengovirus Zn infection induced a strong IFN-β response activation in DBA/2J, demonstrating their ability to produce IFN-β locally in the heart (S1 Fig). Moreover, others inflammatory cytokines levels were increased during CVB3/28 infection, without correlation with CVB-TD viral loads (S2 Fig).

In order to understand the link between RNA domain I structures and IFN-β signaling pathway induction, we investigated the direct impact of CVB-TD and CVB-FL populations mix *in vivo*. Inoculation of homogenized mouse hearts from 14 and 28 DPI in mice revealed that CVB-TD populations without CVB-FL forms replicate in hearts and induce myocarditis without an IFN-β production increase (Fig 2B and 2C). Moreover, replicative 8-36nt and 37-50nt CVB-5’TD forms remained detectable in hearts long after the infection and could be persistent forms (S3 Fig). We then explored how these low-level replicating RNA forms could escape the immune sensors and induce a persistent infection.

Using a strategy based on the transfection of synthetic CVB3/28 RNA mimicking mouse emerging 5’terminal deletions into mouse and human cardiac cells, we studied the direct impact of CVB-FL and 5’TD RNA forms on IFN-β signaling pathway and myocarditis pathogenesis (Figs 3 and 4). Remarkably, *in vivo* transfection of CVB-5’TD and FL forms revealed that 5’TD21 and 5’TD50 RNA forms can induce inflammatory foci, without IFN-β increase in the heart (Fig 3). This result demonstrates the direct cardiac pathogenicity of these two major CVB-5’TD forms, and their ability to escape innate immune clearance in immunocompetent mouse. In human cardiac cells, we identified stem-loops “b”, “c” or “d” of the viral RNA domain I (cloverleaf) as essential viral genomic structures responsible for IFN-β pathway induction (Fig 4). To date, whether the RLR recognition is dependent of RNA sequences or secondary structures remains unknown. We aim to determine the major intra cytoplasmic innate immune sensor (RIG-I or MDA5) responsible for CVB 5’UTR detection using knock-out mice or cells for each RLR and to identify viral sequences and RNA secondary-structures that specifically interact with RIG-I or MDA5.

Since we demonstrated that early-emerging CVB-5’TD forms modulate IFN-β signaling pathway activation, we studied the impact of Poly(I:C), a synthetic dsRNA known to induce type I IFN response through TLR3 (Toll-like-receptors 3) and RLR, on CVB3/28-induced myocarditis in DBA/2J mice (Fig 5). We showed that IFN-β signaling pathway activation at the onset of CVB3/28 infection decreased viral replication and translation levels, and the cardiac cells necrosis (Fig 5). IFN-β levels in the heart of infected and treated mice were elevated, in comparison with infected non-treated mice, associated with a significant increase in overall survival (Fig 5). This result highlights the potential therapeutic benefit of immunotherapies in acute or chronic EV-induced myocarditis.

Altogether, these results indicate that modulation of IFN-β by emerging CVB-5’TD forms could be a key step to achieve long-lasting viral genome maintenance in cardiomyocytes, driving the development of CVB3 acute and persistent cardiac infections. IFN-β signaling is known to directly impair picornavirus replication by inducing RNAse L and 2-5(A) synthetase expression [16]. The 2-5(A) synthetase/RNAse L pathway, exhibiting ribonuclease activity on double-strand RNA, has been shown to be the major mechanism used by IFN-β to inhibit the replication of picornaviruses [16]. Viral populations with 5’terminal deletions in RNA domain I disrupting secondary RNA structures could avoid immune restriction by low IFN-β secretion. Modulation of RLR or TLR3 activation by loss of 5’terminal RNA structures of CVB-5’TD could impair type I IFN pathway activation in infected cells. This mechanism could be a potential way to overcome antiviral immune host response and to lead to the development of sub-acute (14 DPI) and persistent (28 DPI) cardiac infection.

In conclusion, our results indicate that major early-emerging CVB-5’TD forms modulate activation of the type I IFN pathway in cardiomyocytes and induce myocarditis in immunocompetent mice. These findings emphasize the role of replicative CVB-5’TD RNA forms as key pathophysiological players in CVB-induced human myocarditis. Moreover, our results reveal that natural deletions affecting secondary structures of 5’terminal CVB3 RNA domain I can impair innate immune sensing mechanisms in cardiomyocytes during early phase of acute myocarditis. Deciphering the molecular mechanisms leading to the early emergence of CVB-5’TD will enable the development of target immunotherapies to restore an efficient antiviral innate immune response and potentially to achieve a viral clearance in acute and chronic EV-B-induced myocarditis.

## Methods

### Ethic statement

Experiments were performed according to recommendations of the “National Commission of Animal Experiment (CNEA)” and the “National Committee on the Ethic Reflexion of Animal Experiments (CNREEA)”. The protocol was approved by the committee of animal experiments of the Reims Champagne-Ardenne (accreditation number 56) and then by Ministry of Advanced Education and research (permits number #37033–2022042914505068). All efforts were made to minimize suffering in accordance with the Guide for the Care and Use of Laboratory Animals of the Direction des Services Vétérinaires, the French regulations to which our animal care and protocol adhered.

### Cells, virus strain and reagents

HeLa229 cells were grown in minimum essential media (MEM) supplemented by 1% Penicillin-streptomycin (PS) (Gibco, France), 1% L-Glutamine and 10% Fetal Bovin serum (FBS) (ThermoFisher, France). Human primary CardioMyocytes (HCM, ScienCell Research Laboratories) were cultured in cardiac myocyte medium supplemented with 1% PS, 0.01% Cardiac Myocyte Growth Supplement (CMGS) and 5% FBS (ScienCell Research Laboratories) at 37°C with 5% CO_2_. HCM were passaged twice a week. HCM from ScienCell Research Laboratories were isolated from human adult heart tissues and controlled. Indeed, expansion and long-term cultures result in the selection of non-HCM cells. We used low-passages cells (less than 7 passages), with morphological control of cardiac cells phenotype. CVB3/28 virus strain was gift from Steve Tracy (University of Nebraska Lincoln, USA)[39]. CVB3/28 virus is a chimeric cardiovirulent strain, previously tested in immunocompetent mice and *in vitro*. Mengovirus WT and Zn strains were gift from Frank Van Kuppeveld (University of Utretch, Netherlands) [29]. VP1 antibody raised in rat was a gift from Vesa Hytönen (University of Tampere, Finland) [40].

### Virus production and titration

HeLa229 cells were seeded at 0.8x10^6^ cells per well of tissue culture platelet (6 wells) and then incubated at 37°C overnight. The next day, cell confluence was evaluated at 1,5x10^6^ cells per well, based on previous experience, cells were then infected with CVB3/28 at a multiplicity of infection (MOI) of 10^−3^ in free-serum MEM. After 48 hours post-infection, the supernatant was collected and then clarified using low-speed centrifugation, virus particles were then measured by plaque-forming-unit assay (PFU). Briefly, HeLa229 cells were infected with CVB3/28, making serial dilutions, for 1h at 37°C. After viral adsorption, the inoculum was harvested, and cells were overlaid with medium containing 2% agarose and incubated at 37°C. After 3 days, viral plaques were visualized using bromophenol blue staining. The viral titers were expressed as plaque forming unit per 1 ml (PFU/mL). The deleted 5’ UTR viral sequences were obtained as described previously [7].

### Infection and transfection of mice

Five-weeks-old male DBA/2J mice, purchased from Charles River laboratory (France), housed under pathogen free condition, were inoculated intraperitoneally with 10^6^ PFU/mice (CVB3/28), 10^4^ PFU/mice (Mengovirus wild-type or Zn strain) and/or 150μg of Poly(I:C) high molecular weight (HMW) in 150μl of saline; uninfected mice were inoculated with 150μl of saline. Mice were treated with 150μg of Poly(I:C) for two consecutive days after a CVB3/28 intraperitoneal infection (one hour and at day 1post-infection). To determine survival rates upon infection, loss of body weight was monitored daily for 28 days post-infection and mice were euthanized if they had ≥ 20% loss of their initial body weight, according to our protocol. Then mice were anesthetized with Ketamine/Xylazin (42.5/5 mg/kg) and euthanized by cervical dislocation before organs collection at various pre-fixed times points post-infection (1, 2, 3, 7, 14- and 28-days post-infection, DPI). Infected or uninfected mice were sacrificed at the indicated times, a complete autopsy was performed, and hearts were taken and fixed in formaldehyde 4% for histology or homogenized in 600μl of PBS solution with TissueLyser LT (QIAGEN, France). For Fig 3, DBA/2J mice were inoculated intraperitoneally with homogenized hearts collected at 3, 14 or 28 DPI from CVB3-infected mice. To obtain purified supernatants of infected hearts, the heart was first snap-frozen, then homogenized in 600μl of PBS solution with TissueLyser LT. The homogenized heart was centrifugated twice at 1000 rpm during 10 min, followed by the collection of the supernatant. *In vivo* transfection was performed by intravenous route using In Vivo-jetPEI reagent (Polyplus Transfection, New York, USA) and mice were sacrificed at indicated times post transfection. The transfection mixture consisted of 100 μg of synthetic viral RNA and 5 μl of In Vivo-jetPEI in 5% Glucose solution. Long-RNA (L-RNA) used as control for irrelevant long RNA, was synthetized from the sequence of a pCI Mammalian vector by retrotranscription using TranscriptAid T7 High Yield Transcription kit (Ref K0441- ThermoFisher) (length of RNA~ 4kb).

### Histology and immunohistochemistry

Hearts were sliced into three transverse biventricular sections as recommended for the diagnosis of myocarditis.[41] For each sample, four-μm-thick paraffin sections were cut at 3 levels. The slides were stained with Hematoxylin, Eosin and Safran (HES). The slides were examined by a pathologist for cellular infiltration, fibrosis, and necrosis. The lesions were graded using a scale of 0 (no necrosis, inflammation, or fibrosis), 1 (1 or 2 foci by slice), 2 (1 or 2 foci on every slice) to 3 (more than 2 foci on every slice). In addition, immunostaining was performed for CD3 (1/1000; Dako A0452), CD68 (1/100; Dako, M0814) and VP1 (1/100; Tampere University, 3A6) as previously described [40,42,43]. Slides were scanned by the CRB (Biology Resources Center) of the Academic Hospital of Reims.

### Total RNA isolation and qRT-PCR

The homogenized cardiac tissues were subjected to digestion with proteinase K (Merck). Total RNA was isolated with TRIzol RNA Isolation Reagents (ThermoFisher, France) method according to the manufacturer’s instructions. RNA were stored at -80°C. Viral RNA copy number was quantified by RT–quantitative PCR with a StepOne plus realtime PCR system (ThermoFisher Scientific) as described previously, using primers amplifying the CVB3/28: sense (5’-CAC ACT CCG ATC AAC AGT CA-3’), antisense (5’-GAA CGC TTT CTC CTT CAA CC-3’) and probe FAM/TAMRA (5’-CGT GGC ACA CCA GCC ATG TTT-3’) (Eurogentec) [44].

### Cytokines quantification

The hearts’ supernatants were used for: 1) measurement of cytokines by Enzyme-Linked Immunosorbent assay (R&D Systems, France) or ELISA kit for mouse IFN-β (MyBioSource, USA); 2) virus particles determination as described above. Viral titers were expressed as plaque forming unit per 1 mg of total protein (PFU/mg). Measurement of total protein concentration in each supernatant was performed by the Bicinchoninate Acid Method (Sigma Aldrich, France) according to the manufacturer’s instructions. Cytokine protein level was expressed as pg or ng/mg of total protein by calculating the ratio cytokine concentration/total protein concentration. For the quantitative determination of human IFN-β concentrations in cell culture supernatants, ELISA assays using commercially available VeriKine Human IFN Beta ELISA kits (R&D Systems, France) were performed according to the manufacturer’s instructions.

### Reverse transcription-quantitative PCR using SybrGreen (RT-qPCR)

RNA was extracted from hearts lysates or cultured cells with TRIzol Reagent (Invitrogen, Life Technologies) and reverse transcribed using SuperScript II Reverse Transcriptase (RT) (Invitrogen Life Technologies) following to the manufacturer’s instructions. cDNA was subjected to PCR using PowerUp SYBR Green Master Mix (2X) (ThermoFisher Scientific) to detect IFN-β. The primers used for PCR detection as follows: for mouse IFN-β, forward primer 5’- GCCTTTGCCATCCAAGAGATGC-3’ and reverse primer 5’-ACACTGTCTGCTGGTGGAGTTC-3’. Primers specific to mouse GAPDH (forward primer 5’-CATCACTGCCACCCAGAAGACTG-3’ and reverse primer 5’-ATGCCAGTGAGCTTCCCGTTCAG-3’) were used as the internal control. PCR was carried out in StepOnePlus Real-Time PCR Systems (ThermoFisher Scientific) programmed as follows: denaturation step at 94°C for 15s, annealing step at 63°C for 10s, and extension step at 72°C for 15s (40 cycles) for human primers or denaturation step at 95°C for 15s, annealing/extension step at 65°C for 1min (40 cycles) for mouse primers, before melting curve. Results were analyzed with ΔΔCT method, where CT is threshold cycle, and normalized to GAPDH mRNA. Data are represented as levels of mRNA relative to that of the mock transfected control samples and are displayed as the means ± SD of results from at least three independent *in vitro* experiments.

### Rapid amplification of cDNA ends-PCR (RACE-PCR) and NGS strategies for CVB-TD detection and characterization

Total nucleic acids extracted from heart tissues were used to generate NGS libraries and RACE-PCR analysis as previously described [6,34]. Total RNA extracted from heart tissues were ligated on the 5’ extremity with Trp1 RNA adaptator using T4 RNA ligase (Ambion) for 24 hr at 16°C. Reverse transcription was generated using Superscript II reverse Transcriptase (Life Technologies) and cDNA was performed on ligated RNA using a specific primer (AvcRev) targeting the 5’ region of the enteroviral. The cDNAs obtained were then used as a template to perform two PCRs with the KapaTaq polymerase (Clinisciences). These PCR steps amplified the 5’ terminal region of ligated viral genomic RNA and added an adapter sequence and barcodes allowing the multiplexing of the samples using primers. The detection and characterization of CVB-TD forms was performed by RACE-PCR assay and then confirmed by NGS. Sequence reads and alignment against the 5’UTR of CVB3/28 were analyzed with a plugging developed by Thermo Fisher Scientifics. Viral populations deleted from less than 8 nucleotides were considered as full-length viral populations [10,34].

### Positive and negative-strand RNA ratio

Negative-strand RNA was isolated from total RNA molecules by annealing a biotinylated negative-strand specific primer (E3REV; 5’-GGAACCGACTACTTTGGGTGTCCGTG-3’) and binding to streptavidin-labeled magnetic beads (Invitrogen, Life Technologies, Saint-Aubin, France). Purified negative-strand and total viral RNA molecules were quantified with a One-Step real-time RT-PCR assay using serial dilutions of the transcripts for the generation of the standard curves. The positive to negative strand viral RNA ratio was then determined using the following calculation: (total EV RNA–negative strand EV RNA)/negative strand EV RNA.

### Single and double-strand RNA ratio

Double-strand EV-B RNA was isolated by digesting single-strand RNA with recombinant bacterial RNase A (ThermoScientific). A mix of 200ng of total RNA with 1μl of RNase and buffer solution of NaCl at 0.3M, were incubated 10min at 25°C. Double-strand viral RNA copy number was quantified with a One-Step real-time RT-PCR assay using serial dilutions of the transcripts for the generation of the standard curves. The single-strand to double-strand viral RNA ratio was then determined using the following calculation: (total EV RNA–double-strand EV RNA)/double-strand EV RNA.

### Replication capacities, progeny and encapsidation assessment

HeLa229 cells were seeded at 1,5x10^5^ cells per well of tissue culture platelet (24 wells) and then incubated at 37°C overnight. The next day, cell confluence was evaluated, based on previous experience, and cells were infected with 300μl of homogenized heart supernatants, for 1h at 37°C. Encapsidation of these viral RNA forms was assessed by challenging viral entry capacity with or without proteinase K treatment (Merck) which digest all proteins, including viral proteins in the sample. After viral adsorption, the inoculum was collected, and cells were overlaid with free-serum MEM and incubated at 37°C. After 72 hours, supernatant and cells were harvested separately. Viral loads and titers were measured as described above.

### Transfection of human cardiomyocytes

Transfection were carried out as described previously [7]. HCM were transfected with various synthetic 8, 15, 21, 50nt deleted and/or full-length CVB3 RNA. Poly(I:C) (HMW) was used as control (#tlrl-pic, Invivogen). Plates were incubated at 37°C for 8 hours.

### Antibodies and western blot

Protein extracts were resolved by SDS-polyacrylamide gel electrophoresis on Bolt NuPAGE 4–12% Bis-Tris Plus gels (Invitrogen, ThermoFisher Scientific; #NW04122BOX) using Bolt NuPAGE MOPS SDS running buffer and transferred to PVDF blotting membranes (Amersham Hybond) with Trans-Blot SD Semi-Dry Transfer Cell. The following antibodies were used: Phospho-IRF-3 (Ser396) (4D4G) Rabbit mAb (Cell Signaling, #4947); IRF-3 (D614C) Rabbit mAb (Cell Signaling, #11904); EiF4G (C45A4) Rabbit mAb (Cell Signaling, #2469) and β-Actin (8H10D10) antibody (Cell Signaling, #3700). HRP-coupled anti-mouse (NA9310V, GE Healthcare) and anti-rabbit IgG, HRP-linked antibody (Cell Signaling, #7074) were used as secondary antibodies. Peroxidase activity was visualized with SuperSignal West Pico PLUS chemiluminescent substrate (#A34580, ThermoFisher Scientific) or SuperSignal West Atto chemiluminescent substrate (#A38556, ThermoFisher Scientific) with the Invitrogen iBright CL1500 Imaging System.

### ELISA assays for double-stranded RNA (J2 based) detection

First, total RNA were extracted by Tri-Reagent assay from heart lysat of DBA/2J transfected with FL, TD50, TD21, TD8 and JetPEI (negative control) for 24, 48 and 72 hours. For the detection of double-stranded RNA (dsRNA), ELISA assays using commercially available Double-stranded RNA ELISA kits (J2 based) (Exalpha Biologicals) were performed according to the manufacturer’s instructions. Poly(I:C) dsRNA was used as positive control to validate ELISA assay and estimate linear relationship between the amount of dsRNA and the read out for the OD450.

### Statistical analysis

Mann-Whitney’s test (2 groups) or ANOVA (more than 2 groups) were used for *in vivo*, *in vitro* data and ELISA results. Kaplan-Meier test was used to assess the statistical analysis for survival rates. Spearman correlation test was performed. The observed differences were considered significant when P values < 0.05. The number (n) of animal used in the experiment is specified in figure legend. All statistical analyses were performed using GraphPad Prism 8 (Prism).

## Supporting information

S1 Fig 5’Terminaly deleted CVB3/28 RNA forms detection and IFN- β production in DBA/2J mice. **a)** Percentage of each 5’TD-CVB RNA forms (deletion position on the genome (nucleotide) detected by RACE-PCR in the heart of infected mice at indicated time post-infection. **b)** IFN- β levels in the homogenized heart supernatants quantified by ELISA at indicated point time post-mice inoculation with CVB3/28, Mengovirus strains (WT), Mengovirus (Zn) or Poly(I:C) (n = 4). Data represent means ± SD (Mann-Whitney U test; *: p<0.05).
(TIF)

S2 Fig The association of the early emergence of 5’terminally deleted CVB populations with inflammatory cytokines response in the heart. **a** IL-10, MCP1 and TNF-α were quantified in the homogenized heart supernatants quantified by ELISA at indicated point time post-infection with CVB3/28 (n = 6). Data represent means ± SD (Mann-Whitney U test; *: p<0.05; ** p<0.001). **b** Linear regression curves and Spearman R coefficient of correlation between IL-10, MCP1 and TNF-α levels and deleted viral forms from 8 to 50nt proportions (%).
(TIF)

S3 Fig Inoculated CVB-5’TD populations remained significantly detectable in the heart and associated with inflammatory cells infiltrates. **a.** EV viral loads following inoculation of CVB-TD cardiac populations into mice, from 1 to 42 days post-infection (n = 4–8). **b.** 8-36nt and 37-50nt CVB-5’TD and FL respective viral loads in mice’s hearts infected with homogenized hearts or CVB3/28, assessed using a RACE-PCR method associated with a micro-electrophoresis from 24 to 72 HPI (n = 4 to 8). **c.** immunohistochemistry for CD3 and CD68 staining. Positive CD3 and CD68 cells were found in inflammatory foci or infiltrates at 7 DPI in the heart of mice inoculated with homogenized hearts or CVB3/28, they were absent in in uninfected mice. Original magnification: heart, 200**×**. **d.** Histological scoring of inflammatory and necrosis infiltrates of 3 slices per condition. DPI: Days Post Infection. HPI: Hours post infection. UI: Uninfected.
(TIF)
