# Supplementary figures and images for: Major Group-B Enterovirus populations deleted in the noncoding 5’ region of genomic RNA modulate activation of the type I interferon pathway in cardiomyocytes and induce myocarditis
Source: PLoS Pathog. 2024 May 2;20(5):e1012125. doi: 10.1371/journal.ppat.1012125 (PMC11093299; doi:10.1371/journal.ppat.1012125)

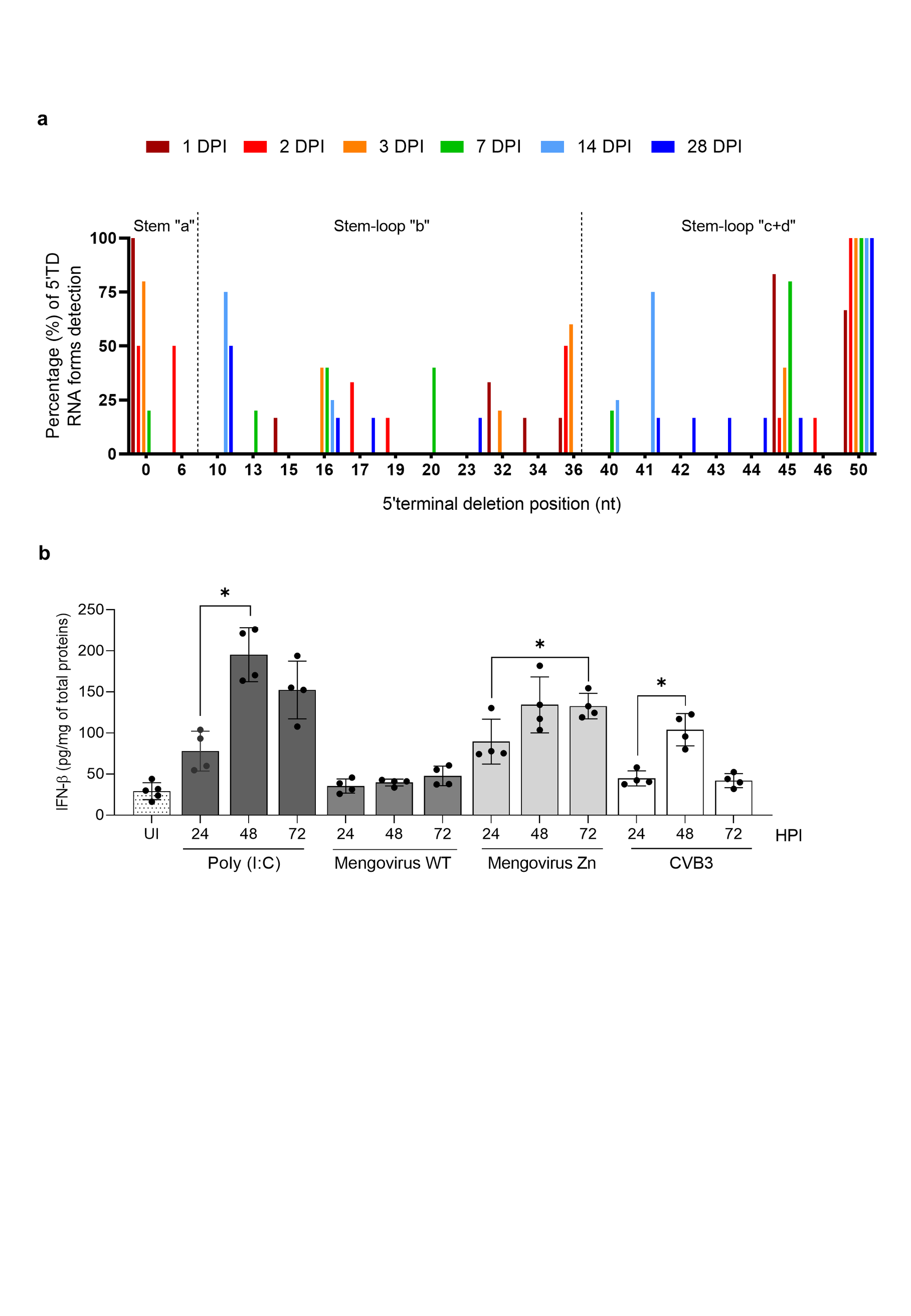

Supplement: S1 Fig — a) Percentage of each 5’TD-CVB RNA forms (deletion position on the genome (nucleotide) detected by RACE-PCR in the heart of infected mice at indicated time post-infection. b) IFN- β levels in the homogenized heart supernatants quantified by ELISA at indicated point time post-mice inoculation with CVB3/28, Mengovirus strains (WT), Mengovirus (Zn) or Poly(I:C) (n = 4). Data represent means ± SD (Mann-Whitney U test; *: p<0.05). (TIF) [file ppat.1012125.s001.tif]

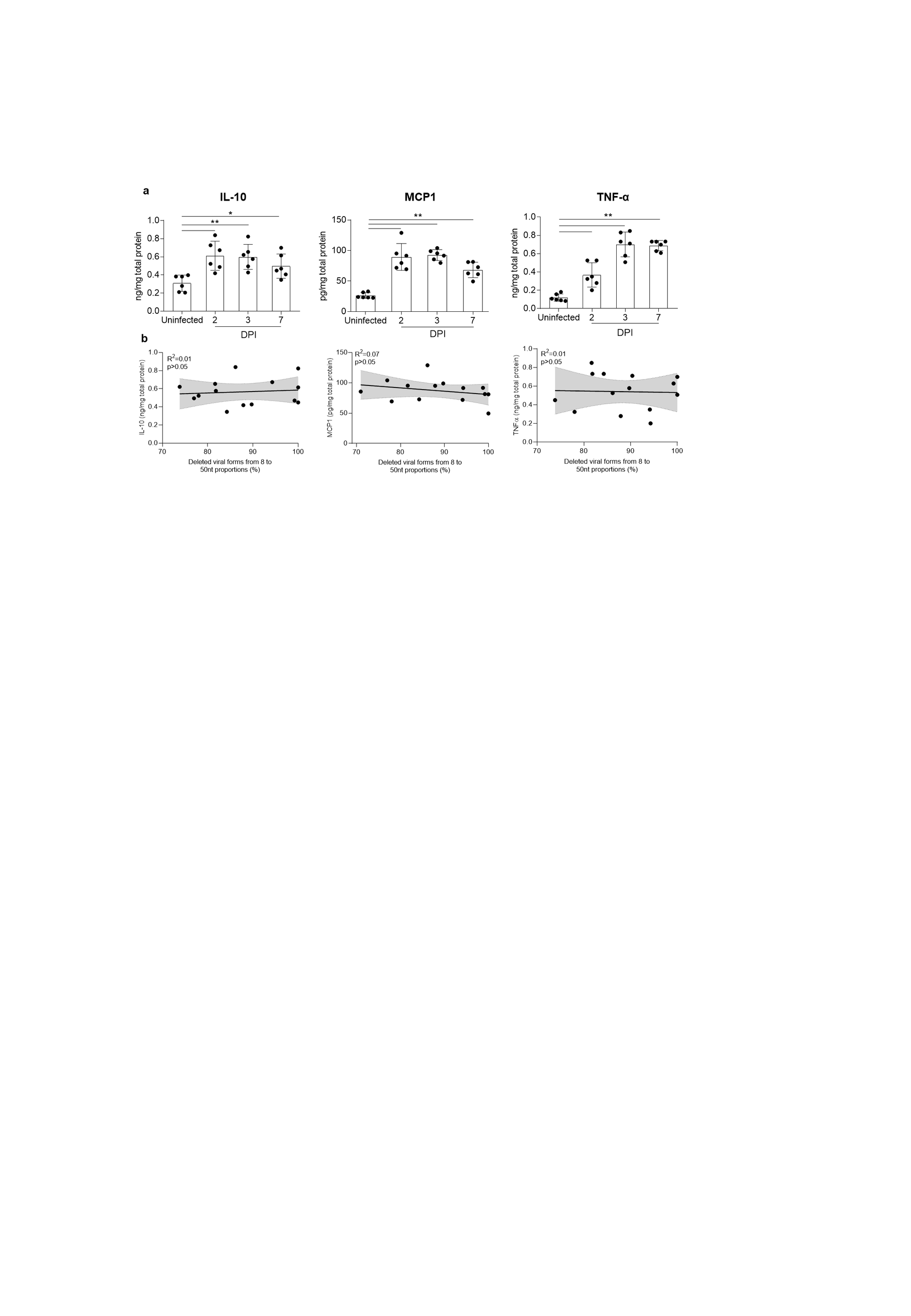

Supplement: S2 Fig — a IL-10, MCP1 and TNF-α were quantified in the homogenized heart supernatants quantified by ELISA at indicated point time post-infection with CVB3/28 (n = 6). Data represent means ± SD (Mann-Whitney U test; *: p<0.05; ** p<0.001). b Linear regression curves and Spearman R coefficient of correlation between IL-10, MCP1 and TNF-α levels and deleted viral forms from 8 to 50nt proportions (%). (TIF) [file ppat.1012125.s002.tif]

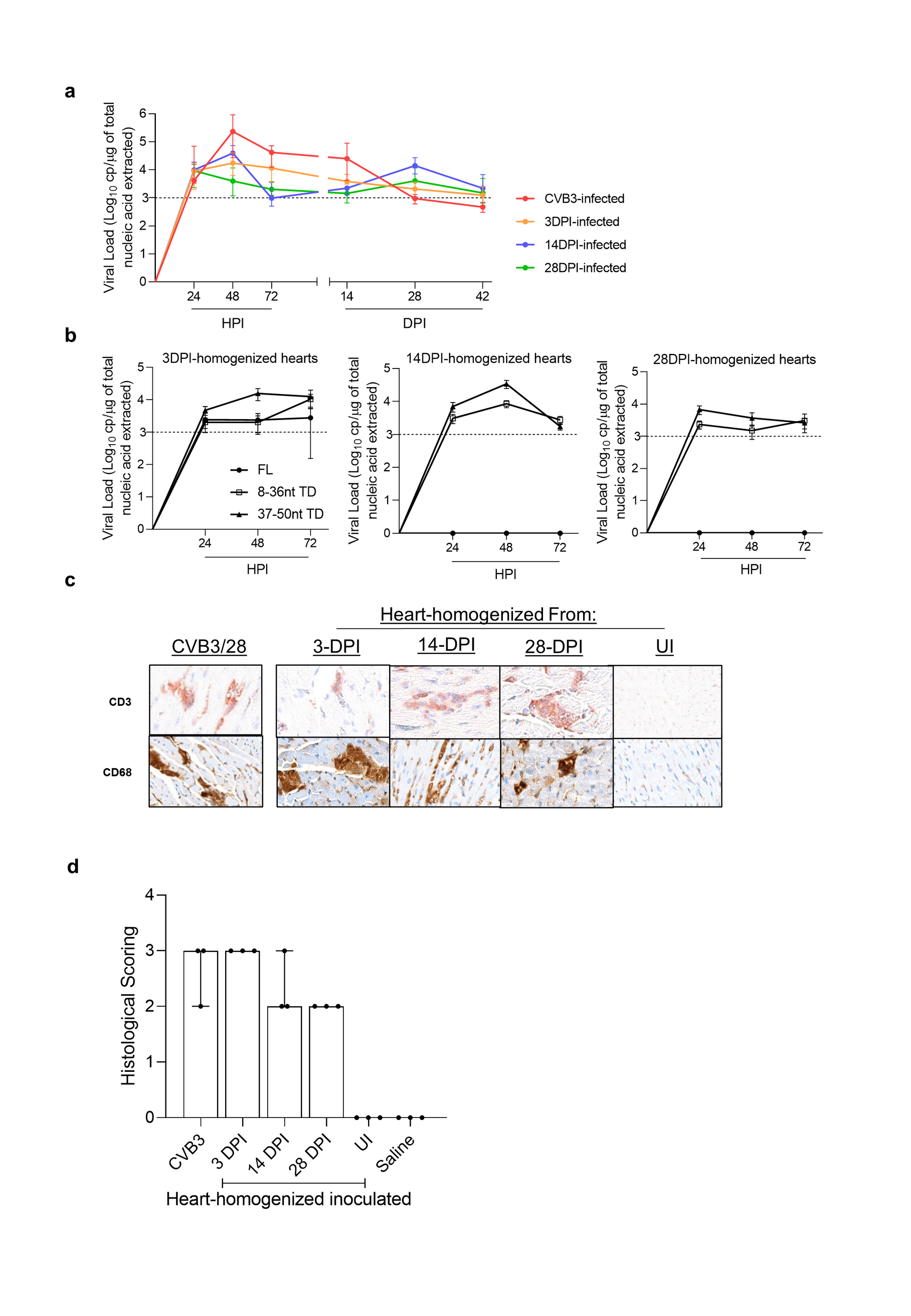

Supplement: S3 Fig — a. EV viral loads following inoculation of CVB-TD cardiac populations into mice, from 1 to 42 days post-infection (n = 4–8). b. 8-36nt and 37-50nt CVB-5’TD and FL respective viral loads in mice’s hearts infected with homogenized hearts or CVB3/28, assessed using a RACE-PCR method associated with a micro-electrophoresis from 24 to 72 HPI (n = 4 to 8). c. immunohistochemistry for CD3 and CD68 staining. Positive CD3 and CD68 cells were found in inflammatory foci or infiltrates at 7 DPI in the heart of mice inoculated with homogenized hearts or CVB3/28, they were absent in in uninfected mice. Original magnification: heart, 200×. d. Histological scoring of inflammatory and necrosis infiltrates of 3 slices per condition. DPI: Days Post Infection. HPI: Hours post infection. UI: Uninfected. (TIF) [file ppat.1012125.s003.tif]
